# Mode-locked ytterbium-doped fiber laser with zinc phthalocyanine thin film saturable absorber

**DOI:** 10.1007/s12200-022-00027-2

**Published:** 2022-06-13

**Authors:** Rawan S. M. Soboh, Ahmed H. H. Al-Masoodi, Fuad N. A. Erman, Abtisam H. H. Al-Masoodi, Bilal Nizamani, Hamzah Arof, Retna Apsari, Sulaiman Wadi Harun

**Affiliations:** 1 Department of Electrical Engineering, Faculty of Engineering, University of Malaya, 50630 Kuala Lumpur, Malaysia; 2 Electronic and Telecommunication Engineering Department, College of Engineering, The American University of Kurdistan, Duhok, 42001 Iraq; 3 Department of Physics, Faculty of Science, University of Malaya, 50603 Kuala Lumpur, Malaysia; 4 Department of Physics, Faculty of Science and Technology, Airlangga University, 60115 Surabaya, Indonesia

**Keywords:** Mode-locking, Ytterbium-doped fiber laser (YDFL), Saturable absorber (SA), Zinc phthalocyanine (ZnPc) thin film

## Abstract

A stable mode-locked laser was demonstrated using a newly developed zinc phthalocyanine (ZnPc) thin film as passive saturable absorber (SA) in ytterbium-doped fiber laser (YDFL). The ZnPc thin film was obtained using a casting method and then inserted between the two fiber ferrules of a YDFL ring cavity to generate mode-locked pulses. The resulting pulsed laser operated at a wavelength of 1034.5 nm having a repetition rate of 3.3 MHz. At pump power of 277 mW, the maximum output power and pulse energy are achieved at 4.92 mW and 1.36 nJ, respectively. ZnPc has a high chemical and photochemical stability, and its significance for use as a potential SA in a mode-locked laser is reported in this work.

**Graphical Abstract:**

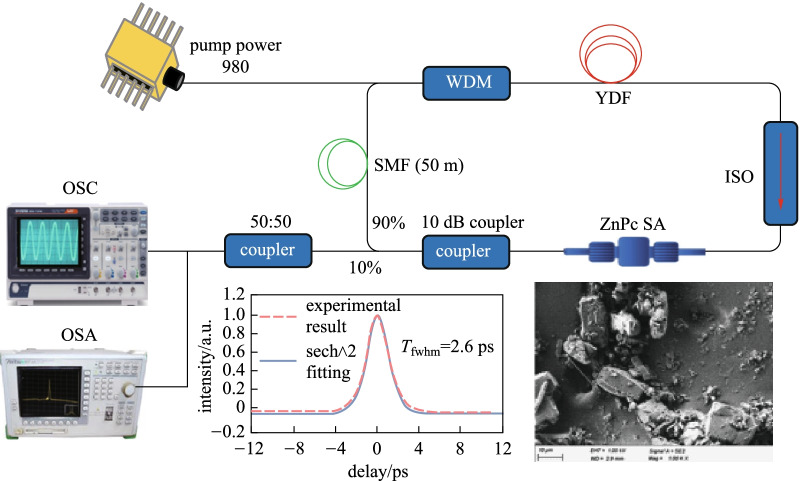

## Introduction

Today, considerable attention is given to optical pulse generation in response to their various implementations in industry, remote sensing and medicine [1, 2]. Mode-locking is one of the methods used to generate ultra-short optical pulsed trains in a fiber laser. Several approaches have been reported to realize the mode-locking, including use of nonlinear optical loop mirror (NOLM) [3, 4], nonlinear polarization rotation (NPR) [5], and the employment of saturable absorber (SA) device. The prime approach is to employ the SA device. It has been employed successfully in different gain media [6, 7]. The operation of mode-locking in 1, 1.5, and 2 µm wavelength regions has been achieved by the utilization of ytterbium-, erbium-, and thulium-doped fibers, respectively [8, 9]. Among these lasers, the ytterbium-doped fiber laser (YDFL) operating in the 1 µm wavelength region has gained much interest in recent years for various communications and sensing applications [10, 11].

Semiconductor SA mirrors (SESAMs) were previously employed as SA due to their benefits of ultrafast recovery time, compatibility with other components, stability, and absorption rate [12, 13]. On the other hand, they have several drawbacks like low damage threshold, high cost and narrow bandwidth, which have limited their applications. In recent years, various types of nanomaterials have been reported for generation of mode-locked pulse train by YDFL. For instance, typical two-dimensional (2D) materials such as black phosphorus (BP) [14], transition metal dichalcogenides (TMDs) [14, 15], and graphene [16] have gained interest in recent years because of their excellent optical and electrical properties. Graphene was widely utilized in various ultrafast pulse lasers [16]. However, its application has been limited due to its small bandgap. Unlike graphene, black phosphorus and TMDs have the desired bandgap that offers opportunities for application in various wavelength regions [14, 17, 18]. However, the performance of BP has been seen to rapidly degrade during its application and it gets damaged easily in a natural environment [19]. Thus, the development of BP is restricted by its mechanical instability. Under these conditions, huge potential for use of TMDs has arisen in ultrafast photonics due to their properties of saturable absorption and third-order nonlinearity [20]. Generally, typical TMDs like MoS_2_- and WS_2_-based optoelectronic devices were largely employed in the visible region, which is determined by their bandgap [20]. However, operation of MoS_2_ and WS_2_ has been reported beyond their bandgap in the near-infrared (NIR) and mid-infrared (MIR) regions [21, 22] due to the broadband property of their saturable absorption. In another research direction, 2D materials such as MXene, antimonene, bismuthene, perovskite, and titanium disulfide (TiS_2_) have also been reported. Well-functioning SAs can be made using MXenes as the main features observed in MXene monolayers can be conserved in stacked ones [23]. Long-term stability and optical response of antimonene and bismuthene based-SAs have also been improved [24, 25]. Perovskite has low lasing threshold and high modulation depth, and thus, it can be used in SA applications [26, 27]. Furthermore, TiS_2_ SA has reduced noise intensity and enhanced signal to noise ratio [28]. Recently, some other nanoparticle-based SAs have been reported in literature, including lead sulfide (PbS) and iron oxide (Fe_3_O_4_) [29, 30]. The nonlinear optical behavior of narrow-bandgap materials is also reviewed for potential applications in pulsed lasers [31]. By modification of conventional optical fiber, tapered optical fibers can be prepared by a flame brushing technique or wheel polishing technique. These tapered fibers can be coated with nonlinear optical materials to be used as SA [32, 33]. The SAs based on D-shape fibers introduce birefringence which allows generation of dark pulses [34, 35]. However, the exposed cladding of tapered fibers makes the system more sensitive to the ambient environment. This method is more suitable for optical fiber sensing applications such as in temperature sensors [36, 37]. Alternatively, polyvinyl alcohol (PVA) based thin films can be prepared and sandwiched between two fiber ferrules to work as SA [38, 39]. This does not require integration of any tapered fiber in the fiber laser cavity.

Recently, organic materials have been introduced as new SA candidates for generation of short optical pulse train due to their fast recovery time, high optical damage threshold, large optical nonlinearity and environmental safety. These organic materials are hybrid organic–inorganic perovskites [40], tris-(8-hydroxyquinoline) aluminum (Alq_3_) [41, 42], and bis[2-(4,6-difluorophenyl) pyridinato-C2,*N*] (picolinato) iridium (III) (FIrpic) [43]. On the other hand, an organic semiconductor, phthalocyanine (Pc) has been extensively investigated for its electronic, photoelectronic, and optical properties [44–49]. Among these materials, zinc phthalocyanine (ZnPc) is a promising organic semiconductor for optical applications.

ZnPc shows high optical absorption in the red-visible region with a suitable optical stability, and thus it is proved to have desirable extinction coefficient in the near infrared region. Since most organic solvents have low solubility [50, 51], ZnPc shows a great potential to be formed as a thin film because of its physical stability. However, the behavior of ZnPc as SA for YDFL has not yet been reported.

In this paper, the ZnPc based SA is employed to generate YDFL mode-locked pulses operating at wavelength around the 1 µm. The SA thin film is formed by integrating ZnPc material into PVA film by using the casting method. By applying the film into the YDFL cavity, a stable mode-locked optical pulse train is obtained. The mode-locked lasing is achieved with a picoseconds pulse width and repetition rate of 3.3 MHz, where the center of the wavelength spectrum is at 1034.5 nm. The un-dissolved particles within polymer spread over the thin film in a uniform manner due to the liquid phase exfoliation, and the high concentration of these un-dissolved ZnPc particles makes the absorbance higher in near infrared region [52, 53].

## Fabrication and characterization of saturable absorbers

The organic material ZnPc and the PVA powder were purchased from Sigma Aldrich. 100 mL de-ionized water and 1 g PVA were mixed and then followed by sonication for one hour at a room temperature using an ultrasonic bath sonicator. Then, 5 mL of PVA solution was poured into 60 mm (diameter) petri dish and the sample was left to dry for three days to form a PVA thin film that could be used as a substrate for the organic thin film. ZnPc solution was produced by mixing 0.5 mL acetone with 10 mg of ZnPc powder, followed with stirring by magnetic stirrer for 30 min at 45 °C in order to produce a homogenous solution. The ZnPc solution was poured onto the PVA layer, followed by drying at a temperature of 45 °C for 30 min to fabricate ZnPc thin film on the PVA layer. The combined ZnPc-PVA film was around 50 µm thick. Finally, a small piece of ZnPc-PVA thin film was attached to fiber ferrules to be used as SA for mode-locking.

**Fig. 1:**
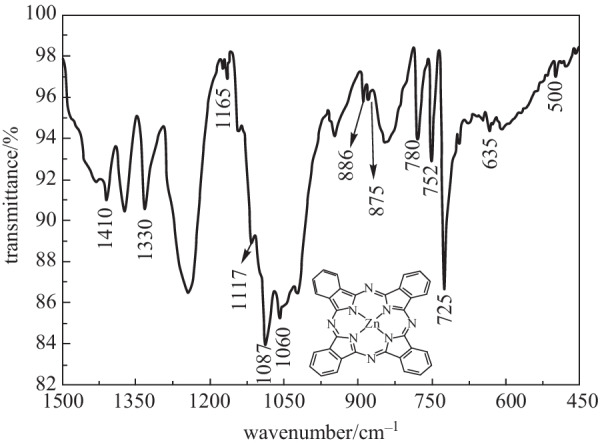
FTIR spectrum of ZnPc thin film. Inset: ZnPc molecular structure

A Perkin Elmer Spectrum 400 Fourier transform infrared (FTIR) spectrometer was used to identify the chemical constituents of ZnPc. The fingerprint region was in the wavenumber range from 1500 to 450 cm^−1^ at a resolution of 2 cm^−1^. Figure 1 shows the FTIR spectrum of ZnPc thin film. The ZnPc is a complex molecule of metal phthalocyanine which is composed of four isoindole units linking with four nitrogen atomswhich are linked by an atom of zinc. The isoindole unit is a benzo-fused pyrrole molecule. The ZnPc molecular structure is shown as an inset in Fig. 1. These molecules appear in the spectrum of FTIR due to vibration in bending and stretching modes. The isoindole stretching vibration peaks were at characteristic peaks of 1410 and 635 cm^−1^. In-plane vibrational peaks of ZnPc molecule are at 1330 cm^−1^ for pyrrole stretch, 1117 and 1087 cm^−1^ for C–H bend, and 752 cm^−1^ for C–H deformation. C-H bend peaks of ZnPc were found at 1165 and 1060 cm^−1^. Out-plane vibration peak of ZnPc molecule for C–H deformation peak appeared at 725 cm^−1^. Zn-N vibration peaks were located at 875 and 500 cm^−1^. Benzene breathing vibrational peaks are at 886 and 780 cm^−1^ [54–57]. The remaining characteristic peaks are related to PVA molecules, PVA being the host polymer for ZnPc thin film [58].

**Fig. 2:**
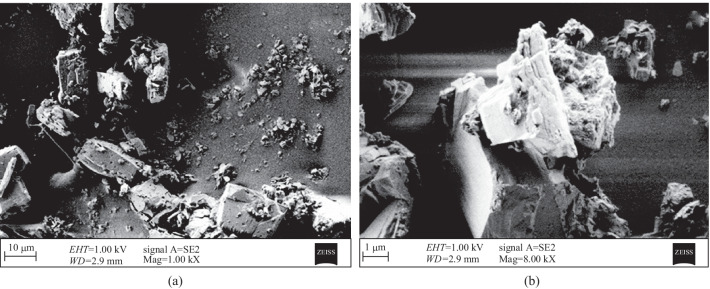
SEM images of casting ZnPc thin film in two different magnifications of **a** 1 kX and **b** 8 kX

The SEM image of ZnPc film on PVA host can be seen in Fig. 2. The surface morphology of the thin film appears as a flat surface with particle aggregations (Fig. 2a). These particles appear as small particles of size of several nanometers and large particles of size of several microns that are due to agglomerations of un-dissolved and dissolved particles of ZnPc molecules, respectively. Figure 2b illustrates the SEM in higher magnification that confirms that the dissolved large particles manifest the crystalline structure of ZnPc powder.

**Fig. 3:**
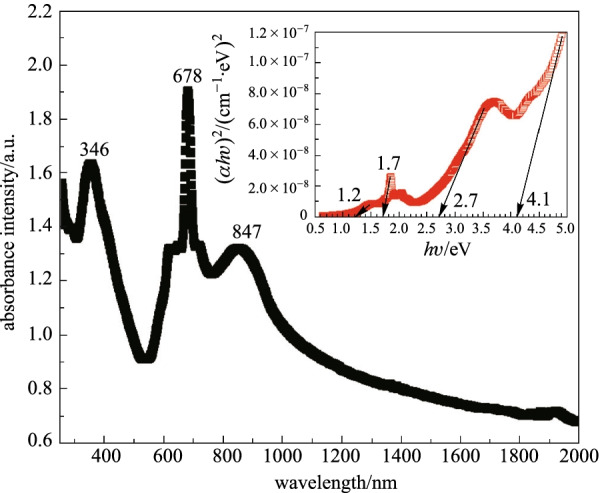
Optical absorption characteristic against wavelength and inserted figure is Tuac’s plot for the bandgap calculation

Optical absorbance spectrum of casting ZnPc on PVA based thin film is demonstrated in Fig. 3, showing three clear peaks. There are two types of energy bands for ZnPc organic material which are Q-band and B-band. The appearance of B-band is at 346 nm while the Q-bands are at 678 and 847 nm. The Q-bands are attributed to $\uppi \to {\uppi }^{*}$ transition energy of the phthalocyanine macrocycle and an excitation state energy [55, 59, 60]. The sharp peak at 678 nm is related to the crystalline ZnPc particles while the red shift in the Q-bands as specified in the literature is attributed to the clustering of the ZnPc particles inside the film [61, 62]. The Beer-Lambert’s law equation $\alpha =2.303\times A/d$ can be used to calculate absorption coefficient $\alpha$, where ZnPc PVA film thickness *d* is about 50 µm and *A* is absorbance. The band gap, ${E}_{\mathrm{g}}$, is calculated from the coefficient of absorption in an equation of ${(\alpha hv)}^{n}=B(hv-{E}_{\mathrm{g}})$ by extrapolating $hv$ to $\alpha =0$. Here, $hv$ is the photon energy, $B$ is a constant (material-related), and *n* is the number of transitions, which is equal to 2. The bulk ZnPc has energy band gaps of 1.53 and 2.97 eV, which are related to Q-band and B-band energy, respectively [59]. Inset of Fig. 3 shows the Tuac’s plot for the bandgap calculation. It is found that the composite thin film exhibited four band gaps at 4.1, 2.7, 1.7, and 1.2 eV, which correspond to modified PVA, B-band and two Q-bands for ZnPc, respectively. Compared to bulk material, the band gaps for the casting ZnPc thin film are slightly shifted due to the dispersion effect induced by the agglomeration of the ZnPc particles inside the PVA film.

**Fig. 4:**
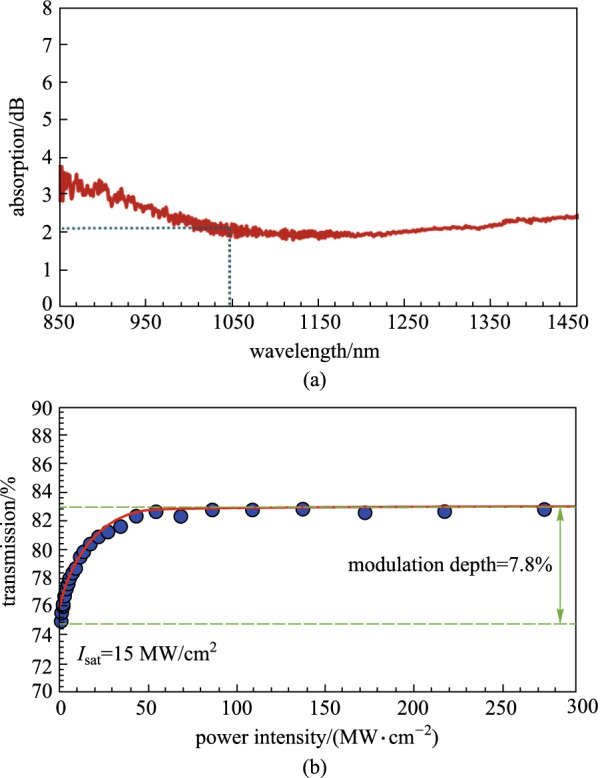
**a** Linear absorption spectrum of ZnPc-PVA SA. **b** Nonlinear transmission property of the casting thin film

The linear absorption of prepared ZnPc-PVA SA was investigated by using a low intensity white light source, and the results were analyzed using an optical spectrum analyzer (Anritsu, MS97010C) with 0.05 nm resolution. As shown in Fig. 4a, the linear absorption of the SA was around 2.14 dB at 1034.5 nm wavelength. By using the balanced two-arm measurement detection scheme, the nonlinear absorption of our prepared ZnPc thin film was measured as plotted in Fig. 4b. 1 μm wavelength mode-locked light source was used for nonlinear optical characterization, which has pulse width and repetition rate of 2.1 ps and 1.8 MHz, respectively. As can be seen from the plot in Fig. 4b, the modulation depth and saturation intensity of the ZnPC-PVA SA are determined to be around 7.8% and 15 MW/cm^2^ respectively, by fitting the experimental data with Eq. (1) [63], where $T$, $\Delta T$, $I$, ${T}_{\mathrm{ns}},$ and ${I}_{\mathrm{sat}}$ are the transmission ratio, saturable absorption or modulation depth, incident laser intensity, non-saturable absorption and saturation intensity, respectively.

1 $$T\left(I\right)=1-\Delta T\times \mathrm{exp}\left(-\frac{I}{{I}_{\mathrm{sat}}}\right)-{T}_{\mathrm{ns}}.$$

## Setup for the experiment

**Fig. 5:**
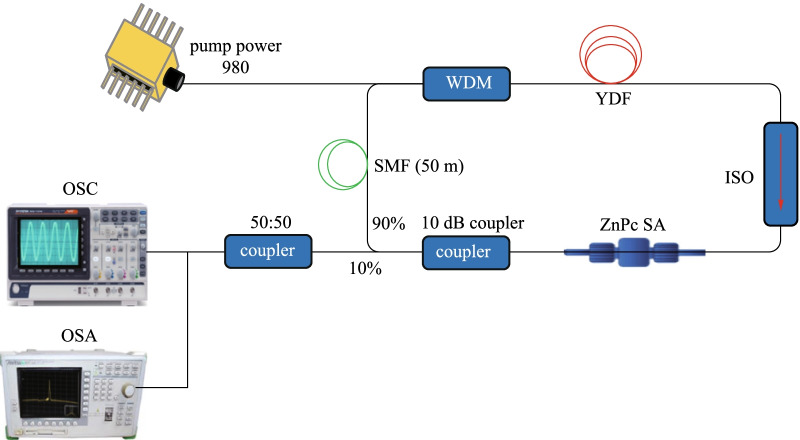
YDFL cavity configuration for the mode-locking operation

Figure 5 illustrates the experimental arrangement of the mode-locked YDFL using the prepared ZnPc SA as a mode-locker. It uses a commercial ytterbium-doped fiber (YDF) as the gain medium and a 980 nm laser diode (LD) as a pumping source. The pumping light was coupled into YDF via a wavelength division multiplexer (WDM). The YDF used had core diameter, cladding diameter, and numerical aperture of 4 µm, 125 µm, and 0.20, respectively. A polarization-insensitive isolator ensured the single directional light propagation in the ring cavity. The SA thin film was inserted between two clean ferrules to form a SA device, which was positioned between the isolator and the output coupler (90:10). The output coupler functioned to allow 90% of the photons to oscillate in the YDFL cavity. The other 10% were tapped out from the coupler to another 3 dB coupler to observe the time domain and the optical spectrum simultaneously. The cavity had a 1.5 m YDF and a 50 m single mode fiber (SMF) with group velocity dispersion (GVD) of 24.22 and 21.91 ps^2^/km, respectively. The entire cavity length including both fibers and other optical components was about 60 m with calculated all-inclusive cavity dispersion of approximately 1.3 ps^2^. Including 50 m long SMF spool in the cavity increased net cavity dispersion which helped to achieve mode-locking instead of Q-switching [64, 65]. A polarization controller (PC) was not added in this experimental setup as the interplay of cavity dispersion and the SA nonlinearity was sufficient to obtain mode-locking operation [66–68]. Hence, a PC was excluded from the experimental setup to avoid any additional insertion loss contribution from the PC. The ZnPc-SA inside the dispersion-balanced cavity caused superposition of propagating longitudinal modes, which as a result generated the mode-locked outputs. It is expected that this kind of mode-locked lasers can produce ultra-short pulses of picosecond to femtosecond duration, with the repetition rate in the range of few MHz.

## Results and discussion

**Fig. 6:**
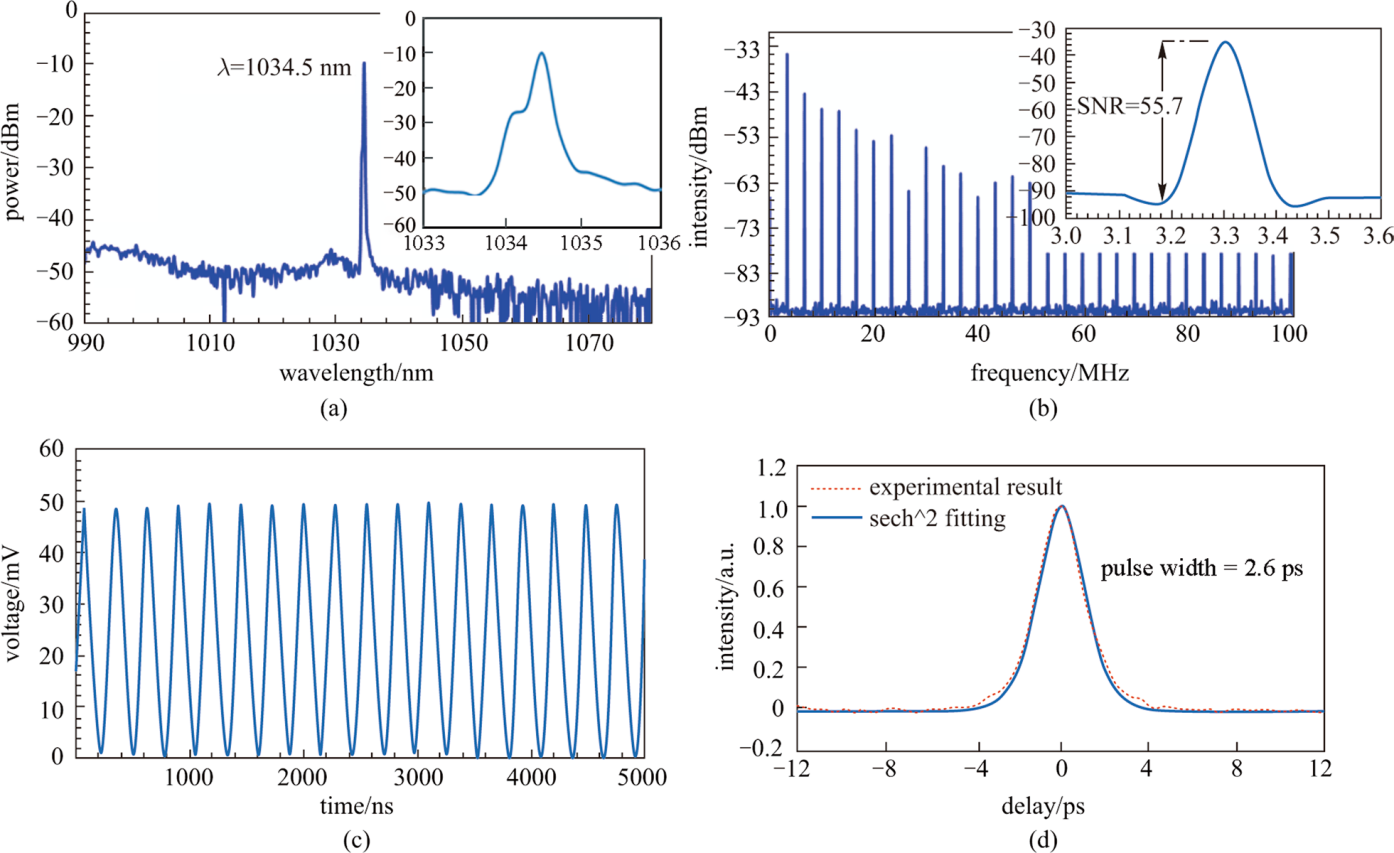
Characteristics of the mode-locked pulses from the YDFL. **a** Optical spectrum. **b** Time domain waveform. **c** RF spectrum at maximum pump power. **d** Autocorrelator trace

The output characteristic of the YDFL before and after insertion of SA was observed by varying the 980 nm pump power. It is worth noting that, at pump power of 150 mW, a continuous wave (CW) laser had been achieved and no pulse had been generated by raising the pump power to the maximum value, before the integration of ZnPc thin film into the ring cavity. Then, after integrating the ZnPc film-based SA between the optical fiber ferrules in YDFL cavity, a self-started and stable mode-locked optical pulse train was generated with pump power changing from the threshold value of 246.3 mW up to the maximum value of 277 mW. The repetition rate of mode-locked pulses was unchanged with the increment in pump power. The optical spectrum of our mode-locked fiber laser output at pump power of 277 mW is illustrated in Fig. 6a. As can be seen, the center wavelength was obtained at 1034.5 nm with 3 dB spectral bandwidth of 0.6 nm without Kelly sideband, which shows the normal-dispersion operation of the laser.

Figure 6b shows the recorded spectrum, which indicates the signal-to-noise-ratio (SNR) of 55.7 dB, at the fundamental frequency of 3.3 MHz. This proved the excellent stability of the mode-locked laser operation. The SNR value can be further enhanced by decreasing the non-saturable loss of the SA and the YDFL cavity. Figure 6c depicts the typical mode-locked pulse train from the YDFL cavity with the ZnPc SA. The pulse train at pump power of 277 mW was recorded using an oscilloscope (GWINSTEK, GDS-3352), which was connected to a 1.2 GHz photodetector (Thorlabs, DET10D/M). It showed a stable pulse train with a repetition rate of approximately 3.3 MHz, which corresponded to peak-to-peak spacing of 276 ns. The repetition rate obtained was well-matched to the cavity length of YDFL, which was around 60 m. The oscilloscope also indicated the pulse width of around 123 ns, which was wider than the estimated actual pulse width because of the limitation of the oscilloscope resolution. Here, the actual pulse width was estimated by using a mathematical model for the time bandwidth product (TBP). Assuming that the pulse follows the Gaussian pulse profile, TBP is equal to 0.441 and thus the shortest possible pulse width is predicted to be around the value of 2.6 ps. The stability of mode-locked pulses could be verified from the radio frequency (RF) spectrum. In this work, the RF spectrum was obtained using an 7.8 GHz RF spectrum analyzer (Anritsu, MS 2803A) in conjunction with a 1.2 GHz photodetector. The pulse intensity profile is monitored by an autocorrelator (AlnairLab HAC-200, resolution < 5 fs). The measured autocorrelator trace in Fig. 6d shows experimental data over sech^2^ fitting which shows the pulse width of about 2.6 ps.

**Fig. 7:**
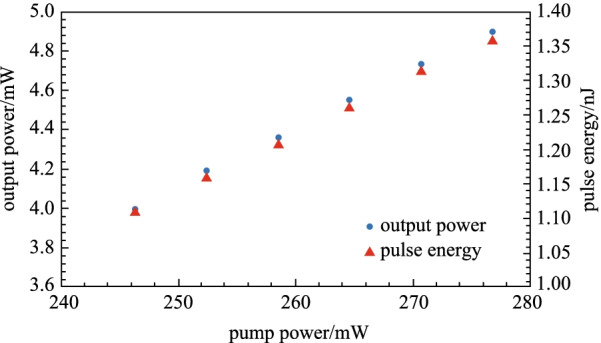
Average power and pulse energy at various pumping power

The relationship between the average output power and pulse energy obtained in the experiment with the pump power is plotted in Fig. 7. It can be seen that as the pump power was raised, both pulse energy and output power were also increased. This is attributed to the larger population inversion induced by the higher pump power, which in turn raised the gain/amplification in the YDFL gain medium. The maximum pulse energy and average power were 1.36 nJ and 4.92 mW.

**Fig. 8:**
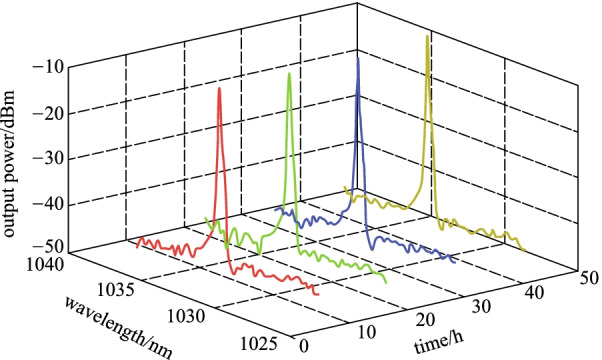
Stability of mode-locked spectra for two days

At a room temperature and under normal laboratory conditions, laser output was maintained at a central wavelength of 1034.5 nm with no obvious shift for at least two days. The mode-locked spectrum with a time interval of 12 h at 277 mW pump power is plotted in Fig. 8, which shows the stability of the laser. The laser operation was maintained at 1034.5 nm without any shift in the spectrum, while the peak intensity was also maitained with a marginal error of ± 0.2 dB.

Table 1 compares ZnPc-SA with 2D materials for the application of mode-locked laser. In the 1 μm wavelength region, the new ZnPc-SA showed great potential for use as a mode-locker. However, there is still room for further enhancement in output power, pulse energy and repetition rate by optimizing the cavity length and the intra-cavity losses. In Table 1, output power of the laser can be seen to be about 4.92 mW, which is slightly higher than that of the mode-locked laser produced by MoS_2_. However, the output power can be further improved if the splicing loss between the optical fiber connecting ends is optimized in the fiber laser cavity. The currently achieved mode-locked laser pulse width is also comparable to the pulse duration of mode-locked Erbium-doped fiber lasers (EDFL) obtained by using TMD-SAs such as WS_2_, few-layer WSe_2_ and MoSe_2_ nanosheets [72, 73].

**Table 1:** ZnPc SA compared with 2D materials for mode-locked lasers at 1 μm wavelength region

SA	Output power/mW	Pulse energy/nJ	Pulse width/ps	Repetition rate/MHz	Operating wavelength/nm	Reference
MoS_2_	2.37	3.1	656	6.74	1042.6	[69]
WS_2_	8.02	2.82	2500	2.84	1030.3	[70]
WO_3_	21.64	2.16	1.67	10	1065	[66]
BP	80	5.93	7.54	13.5	1085.58	[71]
ZnPc	4.92	1.36	2.6	3.3	1034.5	This work

## Conclusion

The generation of mode-locked pulses by using ZnPc-PVA SA was experimentally realized in YDFL for the first time. A self-started mode-locked laser operated with pump power of 246–277 mW, with the center wavelength being located at 1034.5 nm. Corresponding to the output power of 4.92 mW, the highest pulse energy was obtained as 1.36 nJ. The laser operation was stable at repetition rate of 3.3 MHz having a picosecond pulse width. The findings indicate that ZnPc, being a non-toxic material, can be suitable as an alternative SA material for mode-locking application in the 1 µm operation region.
